# The Application of Nanogels as Efficient Drug Delivery Platforms for Dermal/Transdermal Delivery

**DOI:** 10.3390/gels9090753

**Published:** 2023-09-15

**Authors:** Panoraia I. Siafaka, Ece Özcan Bülbül, Mehmet Evren Okur, Ioannis D. Karantas, Neslihan Üstündağ Okur

**Affiliations:** 1 Department of Life Sciences, School of Sciences, Faculty of Pharmacy, European University Cyprus, 2404 Nicosia, Cyprus; 2 Department of Pharmaceutical Technology, Faculty of Pharmacy, Istinye University, 34010 Istanbul, Turkey; ece.bulbul@istinye.edu.tr; 3 Department of Pharmacology, Faculty of Pharmacy, University of Health Sciences, 34116 Istanbul, Turkey; mehmetevren.okur@sbu.edu.tr; 4 Apostolos Loukas Medical Centre, 2415 Nicosia, Cyprus; karantasioannis@gmail.com; 5 Department of Pharmaceutical Technology, Faculty of Pharmacy, University of Health Sciences, 34668 Istanbul, Turkey; neslihanustundag.okur@sbu.edu.tr

**Keywords:** nanogels, dermal, transdermal, delivery, skin, responsive, cross-linking

## Abstract

The delivery of active molecules via the skin seems to be an efficient technology, given the various disadvantages of oral drug administration. Skin, which is the largest human organ of the body, has the important role of acting as a barrier for pathogens and other molecules including drugs; in fact, it serves as a primary defense system blocking any particle from entering the body. Therefore, to overcome the skin barriers and poor skin permeability, researchers implement novel carriers which can effectively carry out transdermal delivery of the molecules. Another significant issue which medical society tries to solve is the effective dermal delivery of molecules especially for topical wound delivery. The application of nanogels is only one of the available approaches offering promising results for both dermal and transdermal administration routes. Nanogels are polymer-based networks in nanoscale dimensions which have been explored as potent carriers of poorly soluble drugs, genes and vaccines. The nanogels present unique physicochemical properties, i.e., high surface area, biocompatibility, etc., and, importantly, can improve solubility. In this review, authors aimed to summarize the available applications of nanogels as possible vehicles for dermal and transdermal delivery of active pharmaceutical ingredients and discuss their future in the pharmaceutical manufacturing field.

## 1. Introduction

The largest human organ, the skin, plays an important role in the defense of the human body. Skin, comprising the epidermis, dermis and hypodermis, has general functions such as to act as a barrier for the external and internal environment as well as to regulate human body temperature and prevent moisture loss [1]. Most importantly, the skin can block microbes from entering the body as well as sunlight and UV radiation. Its main action, protecting the body against penetration of toxic substances including drugs and other molecules is the main obstacle that should be overcome by researchers [2]. Considering the large area covered by skin, the administration of drug molecules via such a route has been widely investigated.

In the last decades, dermal and transdermal delivery methods have gained the interest of medical research society due to the limited side effects, the controlled drug delivery profile, and targeting of skin or systemic diseases [3,4,5,6]. Indeed, dermal delivery, especially for wound healing applications as well as fungal, bacterial and allergic infections, is more efficient than the per os drug administration [7,8]. Various research has demonstrated that topical delivery vehicles such as ointments (creams, gels, pastes) [9], sponges [3], foams [10], films [11] and membranes [12,13,14] present more advantages than disadvantages. More specifically, dermal drug delivery offers rapid medication delivery to the needed site of action, improved local bioavailability as well as patient compliance. However, some of the disadvantages such as skin irritation or low penetration have been managed by employing biocompatible materials and penetration enhancers. In addition, nanotechnology-based carriers have demonstrated better dermal delivery performance than the conventional ones [15]. Nanocarriers are applied for various biomedical applications, including diagnosis, therapy, or both of them as well as in the tissue engineering field [16,17,18]. Polymeric nanoparticles [19,20,21], inorganic nanocarriers [22,23,24], lipid nanocarriers [25,26], microgels [27] and the paradigm of nanogels [17,28,29], which are discussed in this review article, have been widely employed in the medical dermal delivery field; nanogels have great drug-loading capacity, better stability and result in minimal skin damage compared to other forms of gels [30,31].

Moreover, transdermal drug delivery has been extensively studied as an alternative option over other administration routes, especially for chronic diseases where frequent drug administration is in demand [32]. In such cases, the active molecules surpass the skin layers and reach the systemic circulation to induce the therapeutic effect; most marketed transdermal products are “patches”, which as dosage forms can deliver appropriate amounts of drug through intact skin, in a controllable manner [33]. The use of transdermal patches can overcome the various issues that arise with oral dosage forms, such as first-pass effect, gastrointestinal irritation, drug instability due to hydrolysis or degradation by the enzymes and acidic stomach environment [33,34,35]. Given that some drug molecules are unable to penetrate the various skin layers, numerous techniques such as iontophoresis, sonophoresis, or microneedle arrays have been used for efficient transdermal delivery [5,36,37]. Furthermore, nanocarriers have also been extensively studied as potent transdermal vehicles; i.e., nano- and micro-emulsions [38,39], nanoparticles [40,41,42,43] as well as nanogels have exhibited promising results and improved transdermal permeation [44,45,46,47]. It can be said that nanogels have been studied for years in the drug delivery field; nevertheless, since the last ten years, more articles can be found through literature for dermal and transdermal delivery. Nanogels are three-dimensional hydrophilic polymeric network structures of nanoscale sizes; they present viscoelastic properties while their internal structure is similar to that of hydrogels [48,49]. Their unique characteristics, such as desirable mechanical and rheological properties, can affect skin retention time and, consequently, the amount of the drug which can be absorbed. Furthermore, nanogels have great stability and spreadability, which are also important properties for a marketed product intended to be applied on the skin. Even more, nanogels can be formulated via various agents and ingredients which can have an effect on the rheological properties, pH and gel chain macrostructure; therefore, nanogels can act as potent carriers for various drug delivery applications [50]. Nanogels can encapsulate hydrophilic and hydrophobic drugs and, besides dermal delivery, have been used for many other administration routes such as ocular [51], topical oral [52], per os [53], vaginal [54] and others.

According to IUPAC and the standard nomenclature, nanogels are defined as gels with particles with a diameter of 1–100 nm [55,56]. Microgels are comprised of particles having a size between 1 µm and 100 nm, while quasi-gels present particles with sizes only slightly larger than 100 nm [57]. It can be said that gels which are comprised of particles <100 nm can be referred to as nanogels; nonetheless, it should be noted that various applications found in the literature may refer to the developed gels as nanogels if the sizes are <1000 nm.

Nanogels are developed when functional monomers are polymerized and then physically or chemically cross-linked. The physical cross-linking can be achieved via interactions such as hydrophobic, electrostatic or hydrogen bonding between the macromolecules [58]. Chemical cross-linking including emulsion polymerization, controlled/living radical polymerization, click chemistry and photo-induced cross-linking is the most versatile technique for nanogels preparation [59]. An additional synthesis method of nanogels is to initiate the synthesis with polymer chains as substrates and inducing intramolecular cross-linking using a chemical cross-linker as glutaraldehyde. Moreover, ionizing radiation is another synthetic approach of nanogels which simply requires the polymer and the solvent, which mostly is water [60,61,62]. The synthesis of nanogels occurs via intramolecular recombination of radicals which are formed along the chain by ionizing radiation action. The most important thing is that radiation synthesis can synthesize and sterilize the nanogel simultaneously, which is significant since nanogels may possess toxicity [60,61].

**Figure 1:**
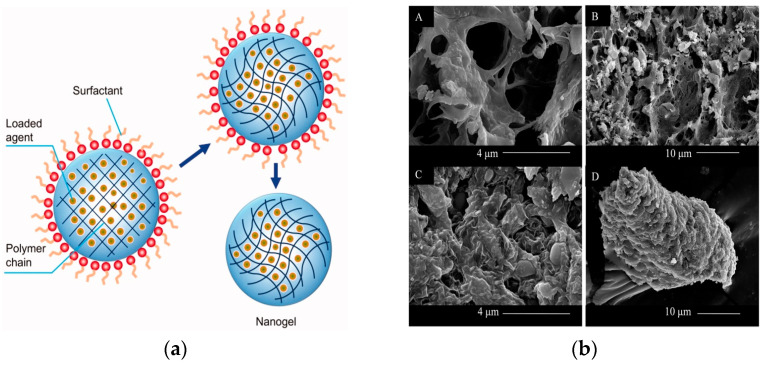
(**a**) Typical nanogel structure developed via inverse emulsion polymerization method. Reproduced by Li et al. (2021), Taylor and Francis under Creative Commons (CC BY) license [67]. (**b**) Morphology of chitosan-based nanogels loaded with myricetin. (A: blank Chitosan (CS)/β-glycerol phosphate (β-GP) gels, 40,000×; B: blank CS/β-GP gels, 10,000×; C: Myricetin-loaded gels, 40,000×; D: Myricetin-loaded gels, 10,000×) Reproduced by Yao et al. (2016) with permission from Elsevier [53].

Although, nanogels can be classified according to their development method as physically and chemically cross-linked, they can also be categorized by the stimuli responsive behavior, i.e., pH-, temperature-, thermo-, ultrasound-responsive nanogels, etc. [63]. The morphology of nanogels can be altered according to the used preparation methods; porous skeleton structure or networks, core–shell structures, etc., have been identified. Figure 1a shows the typical nanogel structure as developed by the emulsion polymerization method and the porous structure of chitosan-based nanogels/gels for oral delivery of myricetin. The most used polymers for the preparation of nanogels are the natural polysaccharides such as chitosan or hyaluronic acid, which are known as biodegradable and biocompatible molecules used in drug delivery [64,65,66]. The synthetic polymers used for nanogels include poly(lactic acid), poly(lactic acid)-poly(glycolic acid) copolymer, poly(methylmethacrylate), poly(N-isopropylacrylamide), and poly(e-caprolactone).

In this review, authors summarized the current applications of nanogels as potent vehicles for both dermal and transdermal delivery. Considering the increased interest of the researchers in fabricating nanogels, this article would be beneficial for the medical and pharmaceutical society as well as students interested in broadening their knowledge on the subject.

## 2. Skin, the Largest Human Body Organ

### 2.1. Healthy and Diseased Skin Anatomy and Its Role as Barrier for Delivery

**Figure 2:**
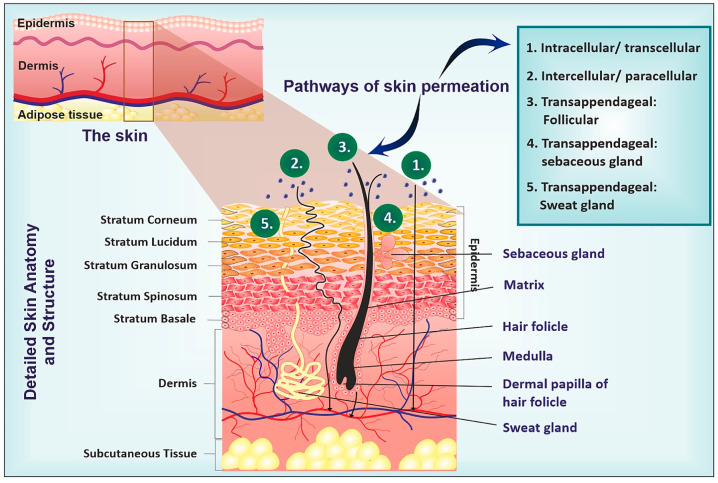
Skin anatomy and permeation pathways. Reproduced by [72] with permission from Elsevier.

The largest organ of the human body is the skin [1,68,69]; 15% of the body weight is attributed to the skin, reaching about 2 m^2^. Skin provides environmental protection and homeostasis maintenance [70], constituting a physical barrier. The massive area that skin covers makes it the perfect candidate for transdermal drug delivery considering also that 33% of blood circulation transpires through the skin. Starting from internally to externally, the three layers of the skin (Figure 2) are the hypodermis, the dermis and the epidermis [71], which is extended between 0.5 and 1 mm, depending on the body area.

More specifically, the epidermis consists of two layers, the viable (stratum lucidum, stratum granulosum, stratum spinosum, and stratum basale) and the nonviable epidermis (i.e., stratum corneum (SC)), which provide an important barrier to environmental elements as well as to most medications and other materials striving to infiltrate the human body through the skin [73]. The composition of this barrier contains hydrophilic keratin proteins compactly packed with hydrophobic lamellar lipids in a unique arrangement. The hydrophobic part of the epidermis is a consequence of its composition of ceramides (50%), cholesterol (25%), fatty acids, etc. [74], as well as the fact that it contains dead corneocytes filled with keratin in a dense arrangement. The lipid bilayer structure and the hydrogen bonding between the components of SC are responsible for the barrier role of SC and unsuccessful drug delivery. SC is responsible for halting any molecules from entering the lymphatic system and drug molecules [75,76]. The various routes through which the environmental elements could penetrate the skin barrier include sebaceous or sweat glands penetration, penetration through the intercellular spaces of the SC, appendage penetration through the hair follicle, transcellular (intracellular) permeation across the corneocytes of SC and using transporter proteins like efflux transporters, which assist in the absorption of large molecules such as P-glycoprotein [5,76]. The viable epidermis, the second barrier, consists of adhesive membrane proteins, tightly adjustive in it. Dermis, as the inner layer between the epidermis and hypodermis, is comprised of connective tissue, blood vessels, oil as well as sweat glands, nerves, hair follicles, and others. Its main action is the strength and elasticity provided to the body due to the elastin fibers and collagen which it is comprised of. The dermis is also responsible for detoxication through the lymphatic system [77]. The last barrier of the skin is the hypodermis, or subcutaneous layer, which connects the skin with inner tissues, i.e., muscles and bones. Its main composition includes routes for nerves, blood vessels and fat cells, which provide heat insulation [78].

The above characteristics of the skin layers are responsible for the difficulty encountered by medications in reaching their target. To overcome this obstacle, researchers are trying to develop innovative drug delivery systems which will effectively deliver the drug through skin barriers. Nanotechnology-based carriers, especially lipid nanoparticles, nanogels, etc., microneedle patches, as well as techniques such as magnetophoresis, sonophoresis, iontophoresis, thermal and laser ablation, are some of the innovative methods [79,80,81,82]. Moreover, chemical disruption of the SC with chemical enhancers, i.e., surfactants, esters, alcohols, terpenes, etc., is also a useful methodology for efficient drug delivery through the skin [83]. Nonetheless, their use (such high doses of enhancers are required) might induce skin irritation and lead to long-term damage of the protective role of the skin due to the disruption of skin lipid structures [84,85].

Although healthy skin can be disrupted via the above methods, damaged skin due to medical conditions may or may not require any method for enhanced permeation. In fact, the existence of any skin medical condition such as atopic dermatitis, eczema or psoriasis could further complicate the dermal drug delivery since these disorders alter the skin barrier.

Various skin disorders such as acne vulgaris, lamellar ichthyosis, psoriasis, Netherton’s syndrome as well as atopic dermatitis are characterized by defective or weakened epidermal barrier functionality, which can be attributed to the altered skin lipid composition and metabolism [86,87]. It has been reported that there is a correlation between lipids in healthy skin and skin diseases with a compromised epidermal barrier [88]. The skin of patients suffering from atopic dermatitis presents decreased content of ceramides in SC, especially of ceramide 1, which differentiates the organization of the lamellar phase and leads to barrier impairment in atopic dermatitis skin. Moreover, cholesterol levels have been found increased in atopic dermatitis skin [89] as well as recessive X-linked ichthyosis. It has been reported that in males with X-linked ichthyosis, the cholesterol sulfate accumulates in the SC, causing abnormalities in epidermal differentiation and the permeability barrier [90]. In addition, an increased content of ceramide 1 oleate at the expense of ceramide 1 linoleate has been detected in dry skin, in skin during the winter season and in essential-fatty-acid-deficient skin [91]. Despite the fact that different ceramide contents have been reported for diseased skin, the lipid lamellae of dry skin or fatty-acid-deficient skin is similar to that of normal skin [91]; nonetheless, in these diseases a reduced skin barrier has been detected. According to studies, the fraction of lipids forming a fluid phase increases, and this excessive presence of a fluid phase results in decreased barrier function. In psoriatic patients, a reduction in the ceramide lipids on the skin surface has been linked with the altered skin barrier [92]. It can be concluded that the lipid profile on the damaged and diseased skin is responsible for the impaired barrier role of the skin [88,93]. Danby et al. reported that the enhancement of SC lipid structure could improve the skin barrier function and protect against irritation in adults with dry, eczema-prone skin [94].

In general, it can be said that the damaged skin could accelerate the penetration of low-molecular-weight, moderately hydrophobic drugs such as corticosteroids and reduce their time residence at the target site; this is important when transdermal delivery is needed. Nonetheless, in the case of dermal delivery and topical wound delivery, the rapid penetration of the molecules would possibly lead to reduced therapeutic outcomes, and efficient carriers which are able to keep the drugs on the skin site are in demand. Nanogels have demonstrated prolonged skin retention and the controlled release of drugs for the desired therapeutic effect.

### 2.2. Dermal and Transdermal Delivery of Drugs

The oral administration of drugs offers significant advantages and efficacy in managing both acute and chronic disease conditions [95]. While they are traditionally used to treat dermatological conditions [96], the preference for oral delivery is diminishing due to higher dose requirements and associated side effects. Consequently, topical products are now increasingly replacing oral medications for localized therapeutic effects [97,98,99]. Notably, the application of drug products on the skin has introduced an alternative delivery approach for drugs which are administered per os; these systems, which are known as ‘transdermal systems’, can induce the permeation of the drug through the skin to achieve the desirable therapeutic drug amount in the blood circulation [100,101]. The increased interest of the research society in dermal and transdermal systems has improved the therapeutic outcomes and resulted in newer and more efficient dosage forms for chronic and acute disorders [102]. At present, researchers and industries have explored strategies such as drug targeting, utilization of modified materials, newer manufacturing processes and characterization techniques to carry out this drug delivery route more efficiently [103]. In the context of dermal delivery, active molecules are administered straight onto the skin at the action site, leading to an increased concentration of the drug in the localized area and a correspondingly lower systemic drug concentration. In contrast, transdermal delivery systems involve the transportation of drugs across the skin surface, allowing them to enter the bloodstream and attain the therapeutic concentrations [104]. The utilization of dermal and transdermal routes for drug delivery offers the advantage of a larger surface area available for drug absorption, facilitating ease of accessibility and providing the flexibility to terminate therapy whenever required. By delivering drugs through the skin, it becomes possible to effectively manage both topical and systemic disorders [105,106].

Dermal and transdermal routes of drug administration are regarded as pain-free methods offering several advantages. They are particularly preferred for the long-term management of chronic conditions such as chronic pain [107], as they provide a continuous and sustained release of medication over an extended period. Moreover, the dermal and transdermal routes bypass the hepatic first-pass metabolism [108], ensuring that the drug is not extensively metabolized in the liver before reaching systemic circulation, thereby enhancing the drug bioavailability and therapeutic efficacy. The transdermal drug delivery system involves the gradual release of drugs through specific layers of the skin which eventually are being transported throughout the entire body via the bloodstream [109]. Despite its advantages, the transdermal drug delivery system also faces several significant challenges, including low drug permeability across the skin, potential local toxicity such as skin irritation, and limitations in penetrating macromolecules such as proteins, peptides, genes, or small interfering RNA (siRNA) [110]. The effectiveness of dermal and transdermal systems is constrained by the skin barriers, particularly the stratum corneum. Consequently, successful drug delivery through the skin necessitates efficient drug penetration across the skin barriers, i.e., lipid bilayer and the keratinized area within the stratum corneum [104]. The application of many existing drugs is restricted due to the need for modifications in their physicochemical characteristics before administration. These modifications are especially necessary for drugs belonging to Biopharmaceutical Classification System (BCS) class II (drugs with high permeability and low solubility) and IV (drugs with low permeability and low solubility), as they exhibit unintended physicochemical properties, such as rapid degradation in the gastric environment [111] and high first-pass metabolism, and could be appropriate candidates for dermal and transdermal administration [112].

## 3. Applications of Nanogels as Drug Delivery Carriers

Nanogels, which are also known as hydrogel nanoparticles or nanoparticles-composed gels, are nano-sized three-dimensional cross-linked (physically or chemically) polymer networks [113,114,115]. Nanogels demonstrate excellent properties, with adjustable size, homogeneity, stability, low toxicity, responsiveness to stimuli (temperature, light, pH, enzymes, etc.), and promising medicine encapsulation capacity [29]. Because of these properties, nanogels are promising for dermal and transdermal preparations and are promising new delivery systems. Dermal- and transdermal-applied nanogels found in the literature are summarized in Table 1.

The nanogels have been widely investigated as potent nanocarriers due to their tunable physicochemical properties. In addition, the nanogels can be modified to have specific particle size, shape and surface charge so as to better penetrate the skin layers. However, such properties may induce the immunological response of the body and should be carefully chosen. In general, key parameters of nanocarriers such as geometry (particle size and shape), chemical nature (composition, surface groups as well as charge, crystallinity, morphology) roughness, porosity, and surface area, surface functionalization (surface coatings, reactivity, and stability), and test media (mostly aqueous) seem to affect the functionality of nanocarriers [116]. According to Anastasiadis et al.’s study, it has been concluded that the dispersion ability of nanoparticles is linked to the chemical composition, surface coating and surface charge, as well as the dispersion media, revealing weak dependence on shape and crystallinity. Moreover, the hydrophobicity/hydrophilicity of nanostructures which has been found to be very significant for their biocompatibility is strongly correlated with the chemical features, i.e., chemistry, surface charge and coating. Furthermore, characteristics such as chemical features, composition, size and surface area and coating, as well as crystallinity affect the dissolution of the nanoparticles, which is also related to the pH and the temperature of the solution. Authors suggested that the antimicrobial activity and biocompatibility of nanoparticles are influenced by the dissolution of nanoparticles. Finally, the physicochemical features of nanoparticles, i.e., size, shape, and surface properties, play a significant in cellular uptake since they control their internalization [116].

Nanogels, meaning nanoparticles hydrogels, are mainly developed by polymers of natural origin, i.e., chitosan, dextran, pullulan, poly-l-lysine, poly(γ-glutamic acid) (γ-PGA), heparin, hyaluronic acid, as well as alginate [117,118]. Other synthetic polymers used for the development of nanogels are PEG, poly(ethylene imine). In fact, the first reported nanogel was based on PEG-PEI. Given that PEI is a toxic material, its modification with PEG improved the biocompatibility of the formulation. The small size (20–220 nm) of PEG-PEI nanogel particles improved their cell penetration, whereas PEI is the ideal carrier for negatively charged molecules due to its high (positive) charge density [119]. Chitosan-based nanogels are widely found in the literature; the positive charge of the polymer influences its dermal delivery due to strong mucin binding [120].

It has been reported that specific sizes of nanocarriers are preferred for dermal and transdermal delivery. For example, the accumulation of nanogels exhibiting small (<100 nm) and rigid core structures is more preferable in the stratum corneum than liposomes, because rigid nanogels can connect within the intercellular space [121]. Sabitha et al. prepared and evaluated topically applied chitin nanogels loaded with 5-Fluoracil for enhanced drug retention within the skin and skin cancer management. The particles depicted mean size of 125–140 nm and a charge of +31.9 mV. It was concluded that the positive charge of chitin strongly interacts with the stratum corneum to loosen the keratin, achieving drug accumulation into the deeper skin layers [122]. Moreover, capsaicin nanoemulgels (advanced form of nanogels) compared to conventional capsaicin-loaded gels revealed better skin permeation, which is attributed to the tunable size and shape of the nanogel particles [123,124]. An in vivo study of cutaneous application of paromomycin entrapped in stimuli-sensitive block copolymer nanogel dispersions for the management of leishmaniasis demonstrated that the nanogels have better antileishmanial ability than pure drug. This can be correlated with the small size of the drug-loaded nanogels (9.19 nm); however, authors have not concluded the small size as a possible factor for the great properties of the developed nanogels [125]. According to various studies, polymeric nanogels which present sizes ranging from 100 to 250 nm can efficiently permeate skin [126,127,128,129].

The surface charge of nanogels can impact the protein binding and cellular uptake. Amphiphilic nanogels, comprised of a hydrophilic polymer matrix which contains hydrophobic groups, have been studied as innovative carriers for hydrophobic drugs. It was reported that by increasing hydrophobicity of the network, increased interactions with proteins and binding as well as interactions of the NGs with cells of the reticuloendothelial system are depicted [130]. Furthermore, Gruber et al. revealed that nanogel amphiphilicity can influence dermal delivery, and, therefore, balancing the network composition by suitable surface hydrophobicity and low network rigidity can enhance dermal penetration [131].

In addition, the physicochemical properties of polymers used for the development of the nanogels can govern the mechanical strength, thixotropic characteristics, and functionality; the features of the polymer are dictated by the monomer chemical nature, molecular weight, methodology, and macromolecular structure [123]. Carbopol 940 is a potent gelling agent, and it has been widely used for the formation of hydrogels, conventional gels and nanogels. According to the study of Algahtani et al., the thixotropic characteristics of the developed nanoemulgels were affected by the Carbopol since both loaded and unloaded nanoformulations showed the same rheological behavior [132]. The molecular weight of the polymer can also affect the dermal penetration of the nanogel; for example, the molecular weight of hyaluronic acid and the use of longer biopolymer chains in the nanogel leads to decreased skin penetration, which could be attributed to the lower mechanical strength of the formulation, insufficient to cross the dermal layer [133].

Moreover, the application of methods such as iontophoresis may improve the skin penetration of nanogels; Toyoda et al. fabricated cancer antigen gp-100 peptide KVPRNQ, demonstrating desirable accumulation of gp-100 peptide and nanogels in the epidermis, and, consequently, Langerhans cell numbers increased in the epidermis [100,121].

**Table 1:** Studies examining dermal and transdermal delivery of active substances.

Active Pharmaceutical Ingredient	Preparation Method	Nano Structure Explanation	Application Route	Application	Evaluation Methods	Ref.
Flurbiprofen	Flurbiprofen was dissolved in the water phase. It was then heated to 60 °C, and the cross-linker was mixed. This aqueous part was then dispersed with the organic phase. Finally, water and dichloromethane were evaporated.	Flurbiprofen-loaded nanogel	Dermal	Drug-free nanogel in HPMC gel, drug-free HPMC gel, and drug-loaded nanogel in HPMC gel formulations was applied for in vivo skin irritation test.	Polydispersity index (PDI), drug content, particle size, zeta potential, pH, visual examination, rheological studies, viscosity, in vivo skin irritation test, permeation, in vitro release, stability	[50]
Meloxicam	Solid lipid nanoparticles (MLX-SLN)-based nanogels containing drugs were studied by microemulsion template technique. Carbopol 940 was dissolved and neutralized by adding triethanolamine.	SLN-based nanogel (SLN-gel)	Dermal	Drug-SLN-contained Carbopol gel and drug-free SLN Carbopol gel were applied for skin tolerance tests and evaluation of pharmacodynamic activity.	Entrapment efficiency (EE), in vitro skin occlusivity, rheological behavior, skin deposition, effect on stratum corneum, in vitro skin permeation, pharmacodynamic activity, skin tolerance	[134]
Aloe-emodin, Acitretin	Chitin nanogels were prepared by regeneration chemistry. The drug solution was added. Remaining steps are centrifugation and sonication.	Aloe-emodin, Acitretin-loaded Nanogel	Dermal	Chitin nanogels, acitretin-loaded chitin nanogels and aloe-emodin-loaded chitin nanogels for evaluation of anti-psoriatic activity and skin irritation study.	Swelling, ex vivo skin permeation, drug retention, in vitro drug release, rheology, in vitro haemolysis assay, cytotoxicity, stability, skin irritation, in vivo anti-psoriatic activity	[135]
Ganoderma lucidum (GLT)	A high-pressure homogenization technique was used to prepare GLT nanosuspensions. Lyofilised GLT nanosuspension was put on the Carbopol 940P mixture.	Freeze-dried GLT nanosuspension powders contained in nanogels	Dermal	GLT nanogel was applied for skin irritation and GLT–Carbopol gel and GLT nanogel were applied for their pharmacodynamic efficacy.	Zeta potential, particle size, drug content, spreadability, pH, in vitro skin permeation pharmacodynamic efficacy	[136]
GLT	A high-pressure homogenization technique was used to prepare GLT nanosuspensions. Carbopol 940 was mixed in water. Nanosuspension and propylene glycol (PEG) were mixed into the Carbopol 940.	GLT nanosuspensions contained gels	Dermal	GLT nanogel was applied for in vitro permeation and placebo gel, GLT nanogel with no therapeutic ultrasound (TUS) and with TUS were used for pharmacodynamic efficacy.	Zeta potential, particle size, drug content, spreadability, pH, in vitro permeation, in vitro release	[137]
Brucine (BRC)	Sodium cholate, lipoid S100, cholesterol and brucine were dispersed with ethanol: chloroform mixture and solvent were removed. The dried, thin film was rehydrated with solution. Mixtures were put in a sonicator to reduce size.	BRC-loaded transliposomes (BRC-TL) contained nanogel	Dermal	BRC-TL, placebo TL and BRC suspension were applied for in vitro cytotoxicity study.	Vesicle size, PDI, drug release, antioxidant properties, EE, pH, firmness, consistency, cohesiveness, viscosity, skin permeation, dermatokinetic study, in vitro cytotoxicity	[138]
Tacrolimus	Ring-opening copolymerization of glycidol and succinic anhydride as a new synthetic production method was studied enzymatically. Novozyme 435 was used for the esterification of oligomers.	Tacrolimus-loaded nanogel	Dermal	Nanogels were loaded with tacrolimus and applied for skin penetration, cell viability	Skin penetration, cell viability	[139]
Dexamethasone	With a new technique, a supramolecular polymer nanogel was designed that uses host–guest interactions between groups of arene and alkyl chains on the hyperbranched polyglycerol backbone.	Supramolecular polymer nanogels	Dermal	Dye-labeled supramolecular assemblies and supramolecular polymer nanogels were employed to conduct a skin penetration study	Degradation, cell viability, skin penetration, drug release	[28]
Hyaluronic acid/β-glucan	HAMA-OVA and SPGMA were dissolved in phosphate-buffered saline (PBS) and put as a photoinitiator into mixtures. These solutions were mixed, and they were cured. Gels were mixed in PBS, then filtered through a syringe filter.	Hyaluronic acid/β-glucan hybrid nanogels	Dermal	The rhodamine B-labeled nanogels were applied for skin penetration.	Particle analysis, cell culture, skin penetration, flow cytometry, polymerase chain reaction	[17]
Lemongrass *(Cymbopogon citratus)* oil	Encapsulation of lemongrass oil was conducted with the ionic gelation technique. Acrylate was added to convert the emulsion into a gel.	Chitosan-encapsulated lemongrass nanogel	Dermal	The chitosan nanoparticles entrapped in acrylate gel were applied for dermal toxicity.	Fourier Transform Infrared (FTIR), TEM, wash durability, encapsulation efficiency, stability, X-ray diffraction pattern (XRD), durability of nanogel against crocking and perspiration, subacute toxicity, Dynamic Light Scattering (DLS)	[140]
Lidocaine	To prepare the nanoemulsion, lidocaine was dissolved in oleic acid, then an emulsifier was added. Water was put into the mixture slowly. Prepared coarse nanoemulsion was sonicated. It was added to the dispersion of Carbopol 940 with a gelling agent.	Lidocaine-loaded nanoemulsion-based nanogel	Dermal	Topical nanogel and conventional gel were applied for in vivo skin safety study.	Particle size, PDI, percent transmittance, thermodynamic stability, refractive index, zeta potential, pH, morphological evaluation, in vivo skin safety, drug content, extrudability, spreadability, drug release, dermatokinetic study, stability	[141]
Methotrexate	Methanol was added to a chitin-saturated dispersion in methanol calcium chloride mixture by mixing, and it was sonicated. Methotrexate was mixed, centrifugated and sonicated.	Methotrexate-loaded chitin nanogel (MCNG)	Dermal	MCNG with a conventional Carbopol gel was applied for in vivo anti-psoriatic studies and toxicity studies.	EE, loading efficiency, in vivo anti-psoriatic activity, drug release, swelling, skin permeation, cell culture studies, subacute toxicity	[142]
Nisin	Nisin and chitosan were dissolved in citrate buffer. Nisin solution was added to the chitosan slowly. The solution was mixed. For electrostatic interactions, 250-watt power was applied to the solution. This solution was to separate unloaded drugs.	Chondroitin sulfate-Nisin nanogels (CS-N NGs)	Dermal	-	Loading efficiency, DLS, swelling, field-emission scanning electron microscopy (FESEM), loading capacity, in vitro degradation, antibacterial activity, cell viability, in vitro drug release	[143]
Temozolomide	Polylactic-glycolic acid (PLGA) and temozolomide were dispersed in dichloromethane and stirred with polyvinyl alcohol (PVA) solution. The coarse emulsion was with a homogenizer. It was evaporated, cross-linked by sodium triployphosphate (TPP), stirred and lyophilized. Temozolomide-encapsulated lyophilized nanoparticles were mixed in the Pluronic F-127 gel system.	Lyophilized drug-encapsulated PLGA-chitosan nanoparticles contained nanogel	Transdermal	-	Scanning Electron Microscopy (SEM), particle size, PDI, Thermogravimetry, Differential Thermal Analysis (DTA), Differential Scanning Colorimetry (DSC), rheology, stability, Transmission Electron Microscopy (TEM), sol-gel fraction, EE, porosity, turbidity, sedimentation rate, stability, ex vivo skin permeation, biocompatibility, in vitro drug release	[45]
Diclofenac sodium	Semisolid gels: PEG, water, diclofenac sodium, Tween 20 and DMSO were mixed, and gellan gum was slowly added to this crease and dissolved. Mineral oil was added and mixed. Calcium chloride, isopropyl alcohol, was added and homogenized.Solid hydrogel film: PEG, water, Tween 20, DMSO and diclofenac sodium were mixed, and gellan gum was added slowly and heated at 75 °C. After the gum was thawed, the temperature was slowly lowered. Isopropyl alcohol was added. Calcium chloride was added as a cross-linker. The hydrogels were poured into solid gels and cured.	Diclofenac sodium-loaded temperature and pH-responsive core–shell nanogel	Transdermal	-	DLS, in vitro release, Attenuated Total Reflectance Fourier Transforms Infrared Spectroscopy (ATR-FTIR), SEM	[144]
Caffeine	Poly(NIPAM-co-AAc) nanogel was performed with an easy emulsion polymerization. A post-production method was used to load the caffeine into the produced nanogel particles. Caffeine was included in lyophilized nanogel with magnetic stirrers. The mixture was ultrasonicized and incubated at 2–4 °C and ~25 °C.	Caffeine-loaded poly(NIPAM-co-AAc) nanogel	Transdermal	Caffeine-loaded poly(NIPAM-co-AAc), caffeine-loaded poly(NIPAM-co-AAc), followed by aqueous solution of pH modulator (CA), caffeine-loaded polyNIPAM, caffeine-loaded poly(NIPAM-co-AAc)-RT, caffeine-loaded poly(NIPAM-co-AAc)-RT, followed by aqueous solution of CA, caffeine-loaded polyNIPAM-RT, saturated aqueous solution of caffeine were used for skin permeation.	Quantitative analysis, particle size, in vitro skin permeation, size distribution, effects of temperature, thermal analysis, pH, TEM, swelling behavior, EE	[145]
Artemether (ART)	For NLC, artemether was mixed at 90 °C, Gelucire, P85G, Transcutol, ethanol added, and Tween 80 added, homogenized with Polytron, and lyophilized. The polymers were dispersed in water, ethanol and PEG were added. The lyophilized NLC formulation was added and mixed well. pH adjusted.	Nanostructured lipid carrier (NLC) contained gel (nanogel)	Transdermal	ART-NLC (1.5 g dispersed in 1 mL of 1:1 water–ethanol mixture, equivalent to 33 mg of ART) was applied for in vivo transdermal anti-plasmodial activity. One gram of ART-nanogels contained 12.5 mg of ART, whereas 1 g of ART-NLC containing 22 mg of ART was applied for skin tolerance test.	Zeta potential, particle size, PDI, size distribution, TEM, DLS, DSC, encapsulation efficiency, in vivo transdermal activity, ex vivo tape stripping, pH, spreadability, in vitro occlusivity, rheology, drug content, skin tolerance, ex vivo skin permeation, in vitro drug release	[146]
Ibuprofen	For polymer–drug nanoconjugates, drug was put in sodium hydroxide and water was added. The drug solution was put into a chitosan dispersion with stirring. Gellan gum was mixed with PEG switch stirring. The drug–chitosan nanoconjugate dispersion was added to the gel at 60 °C.	Ibuprofen–chitosan nanoconjugate contained gel (nanogel)	Transdermal	-	Conjugation efficiency, FTIR, SEM, DSC, rheological studies, thermal gravimetry analysis, pH, swelling, ex vivo skin permeation, drug release, skin retention,	[47]
*Nigella sativa* oil, atorvastatin	Chitosan was dispersed in acetic acid solution, and PVA was dissolved in distilled water, which was then mixed to form the water phase. Span was mixed with ethanol, and Sativa oil and atorvastatin were dissolved in ethanol. This was put into the aqueous part to obtain a microemulsion. It was homogenized for nano size. The solution was stirred while the organic phase was evaporated. Cross-linking was achieved by dripping TPP solution into the emulsion. CMC was added and mixed.	Atorvastatin-Nigella sativa oil-loaded nanogel	Transdermal	Oil nanogel and atorvastatin-oil nanogels were used for in vitro skin permeation tests.	Particle size, zeta potential, FTIR, drug loading efficiency, drug release, viscosity, storage, antimicrobial assessment, in vitro cytotoxicity, in vitro wound closure, gene expression analysis, in vitro permeation	[147]
Gp-100 peptide KVPRNQDWL	The peptide mixture was put into a nanogel dispersion and stirred. pH 6.5 buffer was put into the mixture. The percentage of the peptide to nano gel was optimized for zeta potential.	Antigen peptide-loaded nanogels	Transdermal	Antigen-loaded nanogels were applied for tumor growth inhibition.	Tumor growth inhibition, immunohistochemistry, confocal laser scanning microscopy,	[100]
Luliconazole	The esterified polymer was dissolved with water on a magnetic stirrer. The drug was added and homogenized, and its macrosuspension was prepared. Nanosuspensions were equipped with Sonicator. Carbopol 934 was dispersed and mixed without adding water to the optimized nanosuspension. pH’ was neutralized. The preservative was added.	Nanosuspension-based nanogel of luliconazole	Transdermal	Formalin (standard), 0.9% *w/v* NaCl solution (control) and nanogel were applied for skin irritation test.	FTIR, nuclear magnetic resonance analysis, XRD, SEM, DSC, in silico studies, PDI, particle size, zeta potential, EE, spreadability, pH, viscosity, stability, skin irritation, drug content, in vitro skin permeation	[46]
Methotrexate	The organic part was obtained by stirring the organic phase, magnesium oil and castor oil. The aqueous part was prepared by separately dispersing the aqueous phase, Glycerol, PEG 400, Tween 80 and water. Drug-loaded nanoemulsion was designed by homogenizing the organic and aqueous phases. The preservative was added. Carbopol 940 was added to the mixture and mixed. pH adjusted.	Magnesium oil integrated Methotrexate nanoemulsion-loaded gel (nanoemulgel)	Transdermal	Control groups (DC; CFA treated group), test 1 (MO-S treated group), test 2 (Mtx-MOS) and MTX nanoemulsion contained nanogel were applied for in vivo anti-arthritic activity	PDI, particle size, zeta potential, pH, EE, stability, ex vivo permeation, pharmacokinetics study, in vitro drug release, in vivo anti-arthritic activity	[148]

### 3.1. Nanogels for Dermal Delivery of Active Ingredients

Recently, we have seen the utilization of nanogels as a delivery system that can be applied topically due to their active substance-carrying properties in the literature. After the characterization of nanogels in this area, the therapeutic effect of the active substance against the related ailment after the topical application was generally evaluated. Studies on this subject are summarized in Table 1.

**Figure 3:**
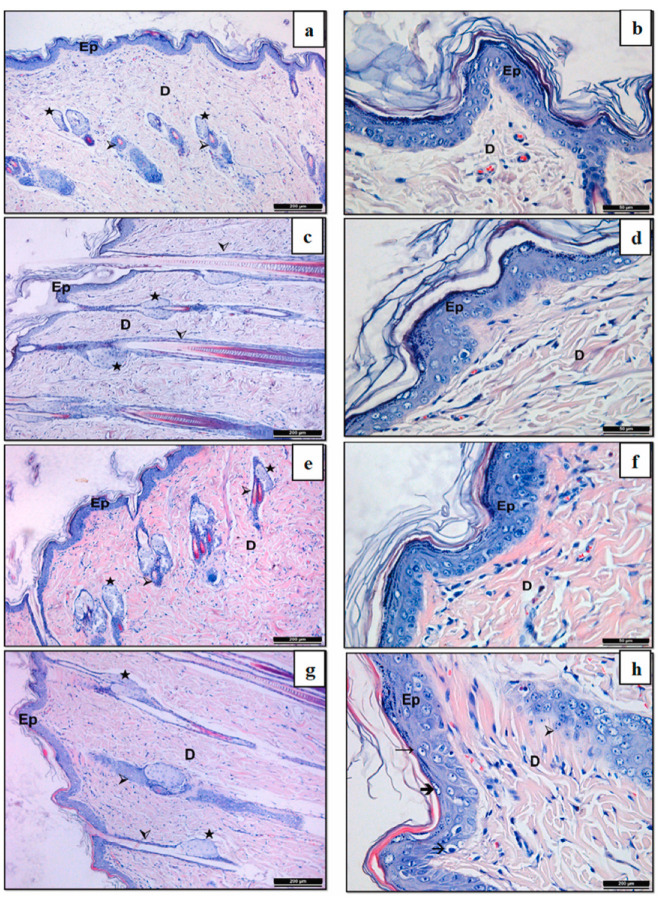
Histological examination of skin irritation studies: Histological findings on skin biopsies from albino rats taken after applying gel formulations. Control (**a**) (100× mag), (**b**) (400× mag); Flurbiprofen -free HPMC gel (**c**) (100× mag), (**d**) (400× mag); Flurbiprofen -free nanogel in HPMC gel (**e**) (100× mag), (**f**) (400× mag), Flurbiprofen -loaded nanogel in HPMC gel (**g**) (100× mag), (**h**) (400× mag). Reproduced by Oktay et al. (2023) with permission from Elsevier [50].

Oktay et al. (2023) developed cyclodextrin-based nanogels for the dermal delivery of flurbiprofen [50]. Authors have further entrapped the nanogels into hydroxypropyl methyl cellulose (HPMC) gel. Authors have studied, both by in vitro and ex vivo models, the developed nanogels in terms of physicochemical and rheological properties as well as skin irritation permeation. According to the results, stable nanogels were prepared with no signs of skin irritation, while permeation studies showed great penetration. Figure 3 demonstrates the histological examination of skin irritation studies. Due to the desirable results, authors should further examine the nanogels via in vivo studies designed for dermal delivery.

Khurana and coworkers prepared a dermally delivered solid lipid nanoparticle gel-based nanogel containing meloxicam (MLX-SLN gel). The authors evaluated the physicochemical, rheological, in vitro permeability and penetration properties and in vivo skin tolerance of meloxicam-loaded nanogels. The nanogel system depicted good skin tolerance and anti-inflammatory activity. When comparing the results with the MLX-loaded nanoemulsion gel (MLX-NE gel) formulated by the authors previously, the flow of the MLX-SLN gel was lower than the MLX-NE gel. Moreover, it has been reported that it can provide controlled and sustained release due to possible drug depot creation in the skin. In addition to extensive in vivo and in vitro experiments, studies with human subjects are needed to understand the usability of the MLX-SLN nanogel in clinical situations [134]. Divya et al. developed a topical nanogel system of aloe-emodin and acitretin with chitin. The particles of the nanogels were spherical, with sizes ranging from 98 to 238 nm, biocompatible, with improved accumulation in dermal layers. The anti-psoriatic activity and skin irritation studies were found to be advantageous in all tests, and the completion of characterization and in vivo tests increased the reliability of this nanogel system [135]. In another study, topical Ganoderma lucidum (GLT) nanogels were formulated to treat frostbite caused by local exposure to extreme cold. GLT nanosuspensions were prepared and then gelled. The superiority of GLT nanogel in the dermal route was compared with GLT-Carbopol gel by performing in vivo pharmacodynamic studies and in vitro skin permeability tests. According to the in vitro permeability studies with rat skin, the nanogel is five times more permeable than GLT–Carbopol gel. In this study, the advantages of the nanogel formulation were demonstrated by in vitro and in vivo experiments in rabbits and rats, highlighting the formulation’s effectiveness [136]. The same research groups subsequently evaluated the effect of therapeutic ultrasound (TUS) on the dermal delivery and frostbite treatment of nanogels isolated from GLT. In addition, the authors showed that TUS is ineffective in drug release from the nanogel, while TUS mainly increases the amount of GLT that permeates the skin. The effect of TUS on the freezing treatment of GLT nanogel was found. This study is a precious and well-thought-out study, as a method was attempted to increase the therapeutic effect of nanogels prepared in the previous year [137]. Alhakamy et al. produced nanogels containing brucine-loaded transliposomes (BRC-TL) that can be applied topically to treat skin cancer. Compared to the BRC suspension in a permeation study, the BRC-TL formulation was approximately 2.5 times more permeable. The accumulation was more significant in rat skin treated with BRC-TL nanogel compared to rat skin treated with BRC conventional gel. In addition to ex vivo and in vitro studies, in vivo studies are needed to demonstrate the treatment ability of the brucin-included formulation [138].

Interestingly, Zabihi et al. created a simple synthetic production method instead of the time-consuming and multistep method. Polyglycerol nanogels were produced by enzymatic ring-opening copolymerization, and Novozyme 435 was used to esterify the produced oligomers to obtain the nanogel. Nanogels entrapped the photosensitizer 5,10,15,20-tetrakis(3-hydroxyphenyl)porphyrin and tacrolimus. It has been reported that nanogels accumulate in the human epidermis (stratum corneum layer), releasing the drug better in comparison to the commercial drug. Here, in addition to a new synthesis method, the advantages of biocompatibility, biodegradability, loading capacity and skin penetration have shown that this method, which shortens the time and reduces chemical consumption, can be used in nanogel production. The reliability of the technique will increase if it can be supported mainly by in vivo experiments [139]. In yet another study, nanogels were combined with supramolecular interactions that were reversible and could easily be processed compared to covalently cross-linked irreversible ones. Dexamethasone, an anti-inflammatory drug, is loaded into nanogels. The supramolecular polymer nanogels have been reported to have a 9-fold increase in skin permeability in a barrier-deficient skin model compared to the conventional cream formulation and individual polymers. To understand the success of the new fabrication method, the authors should further examine nanogels designed for dermal application by in vivo studies [28].

In one study, Kim et al. provided topical delivery of β-glucan/hyaluronic acid nanogels; ovalbumin-conjugated methacrylate-hyaluronic acid and methacrylate-schizophyllan nanogels were prepared. Nanogels with 100–300 nm particle sizes were accumulated in the dermiş by surpassing the stratum corneum of porcine. Transmission is provided to immune cells in the skin. Although the developed system is reported as a potential application for transdermal immunomodulation and vaccination, it needs to be supported by further studies with potential active substances [17]. Kala et al. developed nanogels containing chitosan nanocapsules, including lemongrass (*Cymbopogon citratus*) oil. Afterwards, the gel was impregnated with the fabric to gain long-term mosquito-repellent properties. The data obtained by Scanning Electron Microscopy and Gas Chromatography–Mass Spectrometry confirmed washing resistance, and after 15 washings, the efficiency was found to be 75% in those containing acrylate and 51% in those without acrylate. It has been revealed that the acrylate used in the formulation provides the thickening of the nanogel, and the combination of nanocoating and acrylate increases the washing durability. It can be said that a different wearable mosquito repellent has been successfully prepared since it did not demonstrate any signs of dermal toxicity in the in vivo study on albino mouse [140].

### 3.2. Nanogels for Transdermal Delivery of Active Ingredients

The skin has a large surface area that is extensively exposed to blood vessels and lymphatic networks, thus providing a unique opportunity for noninvasive drug delivery [47]. The transdermal route exists as an alternative route to oral or parenteral administration for long-term, low-dose systemic delivery of drugs. In this way, localized subdermal delivery of drugs is provided. The transdermal route provides lower systemic drug exposure, which prevents gastrointestinal and hepatic first-pass metabolic degradation, which reduces side effects. However, the natural barrier properties of the skin that protect our body are a barrier to drug delivery. The need for the development of innovative formulations and ideas that will increase drug delivery while ensuring drug release in a continuously controlled manner is increasing day by day [144]. The formulations prepared by nanoparticles gel matrix showed a significant improvement in active ingredient penetration in transdermal systems [149]. Here, their use, preparation methods, evaluations and applications in the pharmaceutical field are examined. Studies about transdermal delivery with nanogels are also summarized in Table 1.

**Figure 4:**
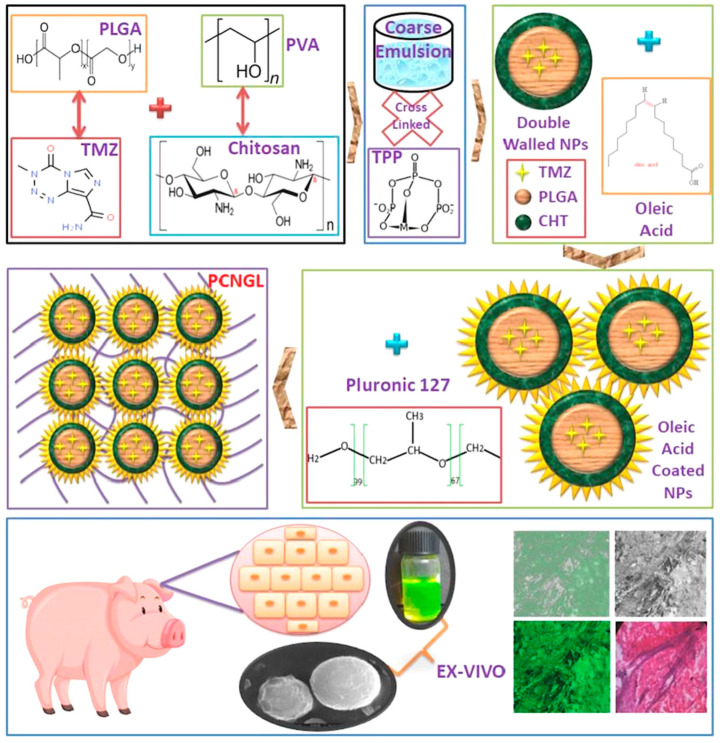
Schematic representation of double−walled PLGA−chitosan surface−modulated nanogel delivery system. Reproduced by Sahu et al. (2021) [45] with permission from Elsevier.

Sahu et al. (2021) fabricated poly(lactic-co-glycolic acid) (PLGA) chitosan double-walled nanogel as an interesting platform for temozolomide transdermal delivery [45]. The developed nontoxic nanogels could be employed as transdermal vehicles according to blood hemolysis and coagulation assays, while in vitro drug release, performed in simulated fluids of dermal microenvironment, demonstrated acceptable release percentages. Finally, ex vivo permeation studies depicted improved penetration and uptake of temozolomide in porcine skin. As in most studies, authors should further explore the nanogels for the pharmacodynamics and pharmacokinetics profile to conclude the effectiveness of the formulations. Figure 4 exhibits a schematic representation of double-walled PLGA−chitosan surface−modulated nanogels.

The essential purpose of transdermal systems is to improve the skin’s permeability. In Carmona-Moran’s study, the increment of the permeability of diclofenac sodium, a nonsteroidal anti-inflammatory drug, using temperature-sensitive nanogels was evaluated. To solve this, semisolid gel and hydrogel film systems containing gellan were prepared, and the effects of penetration enhancers (isopropyl alcohol, propylene glycol and dimethyl sulfoxide) on the permeability of active substance were evaluated. The flow of active substance was 30 μg/cm^2^h from an existing diclofenac sodium topical gel, 44 μg/cm^2^h from the solution formulation, while 130 μg/cm^2^h and 108 μg/cm^2^h from the prepared gel or film, respectively. This study is critical in terms of providing long-term active substance transport from temperature-activated semisolid gel and solid hydrogel film formulations and increasing the transported amount [144]. A temperature- and pH-sensitive nanogel was prepared in another study. The researchers prepared poly(NIPAM-co-AAc) nanogels with temperature- and pH-sensitive poly(N-isopropylacrylamide) (polyNIPAM), copolymerized with acrylic acid (AAc). The loading of the active substance was carried out in deionized water at 2–4 °C and 25 °C, and it was found to be higher at 2–4 °C than at room temperature. Importantly, it has been found that when the poly(NIPAM-co-AAc) polymer is loaded at low temperature, the thermal stimulus of 32 °C created by the skin will initiate the release of charged material. It was also revealed that the nanogels loaded at low temperatures increased the in vitro permeability 3.5 times more than saturated caffeine solution. It was shown that the effect of citric acid, which is a pH modulator, on the release was insignificant [145].

Nanogels have been designed for the transdermal delivery of active ingredients into systemic circulation; one of the most widely used variations of artemisinin in treating malaria is artemether. Nnamani and coworkers prepared nanostructured lipid carrier (NLC) formulations containing ART. After the preparation of the NLC, three polymers (Poloxamer 407, Carbopol 971P, and Prosopis africana peel powder) were used to formulate transdermal nanogels. Nanogels prepared from Poloxamer 407 generally showed superior drug permeability, swellability, pH, viscosity, spreadability and transdermal antiplasmodic properties than Prosopis africana and Carbopol. As emphasized by the authors, it will be possible to declare that it can cure malaria 100% only when it is supported by preclinical and clinical studies [146]. Abioye et al. developed a gellan–ibuprofen–chitosan nanogel for controlled transdermal delivery of drug. Triple nanogels were formulated by ionic gelation and electrostatic nanocoupling. Chitosan increased the skin permeability, penetration and transdermal release rate of drug 4-fold, determined by the extent and concentration of the drug–chitosan ionic interaction. Although ibuprofen appeared to be released by diffusion from pig skin, matrix erosion and drug cleavage occurred as well [47]. In one study, nanogels were loaded with a double-active substance. Atorvastatin and Nigella sativa oil have antioxidant, anti-inflammatory, and antibacterial properties that are useful for wound healing. In another study, Bagheri et al. prepared chitosan–carboxymethyl cellulose nanogels loaded with atorvastatin and black seed oil. These nanogels were reportedly 193 nm and showed that flow through the layers was seen in in vitro skin penetration. Wound healing was confirmed in the in vitro wound closure assay, mainly due to the proliferation properties of fibroblasts whereas the formulation showed bactericidal effects against Staphylococcus species. Further clinical and experimental studies are needed to advance this helpful work [147].

In some studies, nanogels have been used for transdermal vaccination. Toyoda et al. aimed to develop a vaccine against cancer by loading the nanogels with the cancer antigen gp-100 peptide and delivering them via iontophoresis. Due to diffusion in transdermal vaccination, it is necessary to accumulate sufficient antigens in the epidermis for adequate exposure to Langerhans cells. In this study, gp-100 peptide accumulated in the epidermis by iontophoresis, the number of Langerhans cells increased, and tumor growth was halted mainly. It appears to be a suitable formulation for vaccination application if supported by other characterization studies [100].

## 4. Conclusions and Author’s Perspectives

In the last decade, nanogels have gained considerable interest by the pharmaceutical technology society as drug targeting systems as well as diagnostic and therapeutic vehicles. Their important high drug-loading capacity, biocompatibility, ability to entrap both hydrophilic and hydrophobic active molecules, and the tunable rheological properties can categorize them as important drug delivery systems for various administration routes. Applications of nanogels for dermal and transdermal delivery have increased in the last decade due to the better mechanical and rheological properties compared to conventional semisolid dosage forms such as gels, pastes, creams and ointments; their applications for skin disorders (dermatitis, cancer, wound healing) and treatment of systemic diseases as autoimmune diseases can widely be found throughout the literature. The use of dermal transdermal nanogels can be elaborated with better modification via agents which include imaging modalities, targeting moieties and biodegradable functionalized polymers. Besides drugs, other molecules such as peptides, proteins, antigens and antibodies have been incorporated in nanogels, proving the versatile nature of such formulation. Despite their promising characteristics, nanogels marketed products are limited and mostly used as cosmeceuticals. Therefore, more aggressive study of nanogels as pharmaceutical products should be done; in vivo testing is required and frequently missing by the current published articles. The clinical translation of nanogels can only be done if the pharmacokinetics and pharmacodynamics profile is studied; therefore, we encourage scientists working on nanogels for dermal/transdermal delivery to evaluate their effectiveness on approved animal models or use in silico screening tools which seem to be similarly efficient. Finally, scientists should closely explore the fabrication methods of nanogels with biological molecules and/or in combination with active ingredients since these are the future of the medical field, especially for autoimmune skin disorders such as eczema or psoriasis and skin cancer. To conclude, nanogels have all the desirable characteristics to act as potent carriers and offer an alternative solution for disease management.

## Data Availability

Not applicable.
